# Hydrogen production by the hyperthermophilic bacterium *Thermotoga maritima* part I: effects of sulfured nutriments, with thiosulfate as model, on hydrogen production and growth

**DOI:** 10.1186/s13068-016-0678-8

**Published:** 2016-12-19

**Authors:** Céline Boileau, Richard Auria, Sylvain Davidson, Laurence Casalot, Pierre Christen, Pierre-Pol Liebgott, Yannick Combet-Blanc

**Affiliations:** Aix Marseille Université, CNRS, Université de Toulon, IRD, MIO UM 110, 13288 Marseille, France

**Keywords:** *Thermotoga maritima*, Hydrogen, Thiosulfate, Productivity, Growth, Yields, Sulfured nutriments, Glucose, Metabolism

## Abstract

**Background:**

*Thermotoga maritima* and *T. neapolitana* are hyperthermophile bacteria chosen by many research teams to produce bio-hydrogen because of their potential to ferment a wide variety of sugars with the highest theoretical H_2_/glucose yields. However, to develop economically sustainable bio-processes, the culture medium formulation remained to be optimized. The main aim of this study was to quantify accurately and specifically the effect of thiosulfate, used as sulfured nutriment model, on *T. maritima* growth, yields and productivities of hydrogen. The results were obtained from batch cultures, performed into a bioreactor, carefully controlled, and specifically designed to prevent the back-inhibition by hydrogen.

**Results:**

Among sulfured nutriments tested, thiosulfate, cysteine, and sulfide were found to be the most efficient to stimulate *T. maritima* growth and hydrogen production. In particular, under our experimental conditions (glucose 60 mmol L^−1^ and yeast extract 1 g L^−1^), the cellular growth was limited by thiosulfate concentrations lower than 0.06 mmol L^−1^. Under these conditions, the cellular yield on thiosulfate (*Y X/Thio*) could be determined at 3617 mg mmol^−1^. In addition, it has been shown that the limitations of *T. maritima* growth by thiosulfate lead to metabolic stress marked by a significant metabolic shift of glucose towards the production of extracellular polysaccharides (EPS). Finally, it has been estimated that the presence of thiosulfate in the *T. maritima* culture medium significantly increased the cellular and hydrogen productivities by a factor 6 without detectable sulfide production.

**Conclusions:**

The stimulant effects of thiosulfate at very low concentrations on *T. maritima* growth have forced us to reconsider its role in this species and more probably also in all thiosulfato-reducer hyperthermophiles. Henceforth, thiosulfate should be considered in *T. maritima* as (1) an essential sulfur source for cellular materials when it is present at low concentrations (about 0.3 mmol g^−1^ of cells), and (2) as both sulfur source and detoxifying agent for H_2_ when thiosulfate is present at higher concentrations and, when, simultaneously, the pH_2_ is high. Finally, to improve the hydrogen production in bio-processes using *Thermotoga* species, it should be recommended to incorporate thiosulfate in the culture medium.

## Background

Today, the amount of energy derived from fossils fuels (petroleum, natural gas, and coal) represents about 80% of the world energy consumption. It is henceforth recognized that their use has induced very serious environmental pollutions. The accumulation of the greenhouse-gas carbon dioxide in the atmosphere and the depletion of fossil fuels, altogether with the high prices and the ever-increasing demand have forced most of the countries to start looking for cleaner and renewable energy sources. Therefore, major research efforts focusing on solar and wind energy, geothermal resources, and energy derived from biomass were undertaken to develop new technologies suitable for industrial use.

In this context, although dihydrogen (H_2_) is not a primary energy source, it is currently seen as a very promising carbonless “energy carrier” which may be used to store energy and provide an efficient alternative to fossil fuels. Up to now, most of the H_2_ is currently industrially produced by steam reforming of natural gas or by alternative processes based on electrolysis or thermolysis of water [1]. However, in the last decade, biological processes employing bacteria for H_2_ production have received a significant and increasing attention [2–11]. Indeed, like with other biofuels such as ethanol, butanol, fatty acids, and methane, bio-hydrogen can be produced by processes using living organisms such as green algae (photolysis of water), phototrophic and anaerobic microorganisms (photofermentation of organic acids), and anaerobic fermentative microorganisms (dark fermentation of organic substrates) [2, 7, 8, 11–19]. Among these, due to the large spectrum of catabolic activities of H_2_-producing microorganisms, dark fermentation is considered as one of the most promising route. The fermentation processes of these microorganisms potentially allow producing hydrogen from renewable energy sources derived from biomass or various carbohydrate-rich waste streams [2, 20–26].

Depending on the microorganism species used, dark fermentations can be performed at either moderate or elevated temperature. In the former case, hydrogen productivities (QH_2_/time) were generally higher whereas higher yields (YH_2_/substrate) were reached in the latter [4]. Moreover, the advantages of high versus low temperatures for the bio-hydrogen production include better pathogen destruction, reduced risks of methanogen or acetogen growth (hydrogen consuming), and less sensitivity of hyperthermophiles to the H_2_ partial pressure [27–30]. However, in order to develop economically sustainable hydrogen-producing bio-processes, both productivities and yields should be significantly increased. Another economic aspect for these bio-processes is the high fresh water requirement, a resource which needs to be preserved. The use of marine microorganisms, which produce hydrogen in seawater from a wide variety of sugars, appears, in this case, to be a promising approach.

*Thermotoga* spp. are hyperthermophile or thermophile fermentative anaerobic bacteria belonging to a deep-branching lineage within the domain Bacteria [31–34]. They inhabit various hot ecosystems, including hot springs, hydrothermal vents, and oil reservoirs [35–37]. Among *Thermotoga* species, *T. maritima,* originally isolated from a geothermal-heated marine sediment at Vulcano, Italy [32], has received considerable interest as potential hydrogen producer [38]. Indeed, *T. maritima* is able to produce hydrogen with high productivity and yield [29, 39] from a wide variety of sugars, ranging from hexose and pentose monomers to starch and xylan polymers [40]. In addition, compared to the other hydrogen-producing microorganisms, Thermotogales, including *T. maritima*, exhibit the highest H_2_ yields, close to the Thauer limit (4 mol H_2_ per mol glucose). For instance, a yield of 4 mol of H_2_ per mol of glucose has been reported by Schöder et al. [39] when *T*. *maritima* growth was limited by glucose, the energy source, and under very low hydrogen partial pressure (down to 1.3% as hydrogen partial pressure). Actually, *T. maritima* harvests energy by glycolysis via the Embden–Meyerhof pathway (EMP) as the main route and via the Entner–Doudoroff (ED) pathway to a lesser extent (about 85 and 15% of consumed glucose, respectively) [39, 41]. The ultimate pyruvate-reduction steps resulted in acetate, H_2_ and CO_2_ (1:2:1 as molar proportions, respectively) as major end-products of glucose fermentation, and in lactate, alanine, and extracellular polysaccharides (EPS) as minor end-products. The production of these fermentation products was shown to depend on culture conditions (culture medium, nutritional or oxidative stress, operating conditions such as pH, pH2, stirring, Eh,…) [35, 39, 42–46]. In agreement with this classical fermentation model, the H_2_ yield is optimal (4 H_2_:2 CO_2_:2 acetate as molar proportions per mol of glucose or other hexose) only when all the glucose is converted to acetate because the lactate, alanine, and EPS productions are bypassing pathways impairing the H_2_ production. Furthermore, it has been reported in the literature that *Thermotogales* reduced sulfur-containing compounds such as elemental sulfur, polysulfide, and thiosulfate to hydrogen sulfide [47–49]. When *T. maritima* and *T. neapolitana*, two very closely related species [50], were cultivated in the presence of elemental sulfur, and with glucose as energy source, final cell yields were significantly enhanced but their growth rate remained unaffected [39, 51, 52]. In addition, it has been shown that, in the presence of sulfur, glucose was not more efficiently used. Indeed fermentation–carbon–product pattern remained similar (e.g., acetate and CO_2_ as major fermentation products and low amounts of lactate), and hydrogen sulfide was produced at the expense of H_2_ [39]. These findings argue that sulfur reduction was not coupled with energy conservation [39]. In addition, it has been found that (a) hydrogen at high partial pressure inhibited the growth of *T. maritima* [39], *T. neapolitana* [16, 29, 53, 54], and other strains of *Thermotoga* [42, 55], and (b) the presence of sulfur stimulated the growth of these bacteria on glucose at high H_2_ pressures rather than at low H_2_ pressures [39]. Finally, these results were consistent with the Huber proposal suggesting that growth stimulation by sulfur reduction in *T. maritima,* and probably other *Thermotogales,* was explained as an electron-sink reaction preventing the accumulation of inhibitory concentrations of the fermentation product H_2_ [32, 35]. In the same way, other studies have shown that, in *T. neapolitana* and *T. maritima,* addition of other sulfured compounds, such as cystine, dimethyl sulfide, and thiosulfate, could also relieve the inhibition power of H_2_ and/or enhanced the cellular growth [48, 49, 51].

With the aim to optimize the growth of *T. maritima* and to better control its nutritional requirements of sulfur compounds, their effects on growth and glucose catabolism were addressed in this report study. After a comparative study performed in serum bottles, thiosulfate was finally chosen as model among the tested sulfured compounds because of its efficiency for *T. maritima* growth and its stability at high temperature. In a concentration range between 0 and 0.24 mmol L^−1^, the effects of thiosulfate on the growth and glucose catabolism were accurately analyzed from batch cultures, performed into a bioreactor, carefully controlled for pH, temperature, and agitation. In addition, in order to prevent the inhibition of *T. maritima* cultures by hydrogen, the bioreactor was equipped with a specific device allowing controlling and maintaining the hydrogen partial pressure in the bioreactor headspace below the critical limit which was determined under our experimental conditions.

## Methods

### Strain and medium for routine cultures

*Thermotoga maritima* MSB8 was obtained from the DSMZ (DSM 3109^T^). The basal culture medium (BM) used for growth was prepared using anaerobic techniques as developed by Hungate and Macy [56, 57]. It contained (per liter): NaCl 20 g, yeast extract (Fluka Biochemical, Spain) 1 g, NH_4_Cl 0.5 g, KH_2_PO_4_ 0.3 g, K_2_HPO_4_ 0.3 g, MgCl_2_ 0.2 g, KCl 0.1 g, CaCl_2_ 0.1 g, and Balch trace mineral element solution [58] 10 mL. The medium was adjusted to pH 7.0 with 1 mol L^−1^ KOH and then boiled and cooled down to room temperature under a stream of O_2_-free N_2_. It was then distributed into 100 mL serum bottles (35 mL of medium) as previously described [59]. After sealing the serum bottles, the gaseous phase was flushed with a stream of O_2_-free N_2_:CO_2_ (80:20%) for 30 min. The medium was then autoclaved at 120 °C for 20 min and stored at room temperature. Before inoculation, the culture medium was supplemented with 0.75 mL of NaHCO_3_ (100 g L^−1^) and 0.75 mL of glucose (1 mol L^−1^). After inoculation with 1 mL of overnight *T. maritima* culture, final concentrations of NaHCO_3_ and glucose in the culture medium were 2 g L^−1^ and 20 mmol L^−1^, respectively. All *T. maritima* cultures performed in serum bottles were incubated at 80 °C. The stock solutions of NaHCO_3_ and glucose were prepared under anoxic conditions as described by Miller and Wolin [59], and stored under N_2_:CO_2_ (80:20%). The glucose solution was sterilized by filtration and the NaHCO_3_ solution by autoclaving (120 °C for 20 min).

### Culture media for the experiments concerning the study of the sulfur compounds

Stock solutions of DMSO, Na_2_S, methionine, thiosulfate, elemental sulfur, and cysteine were sterilized by filtration and distributed into 100 mL serum bottles. Anoxia was obtained by flushing the bottle headspaces with an O_2_-free N_2_ gas stream for 30 min. DMSO, Na_2_S, methionine, cysteine, and elemental sulfur stock-solution concentrations were 14 mmol L^−1^ and thiosulfate stock-solution concentration was 7 mmol L^−1^. To study sulfur compounds, 100 mL serum bottles, containing 19 mL of basal culture medium (BM) prepared under O_2_-free N_2_:CO_2_ (80:20%), were used. Before inoculation, the culture medium was supplemented with 0.45 mL of NaHCO_3_ (100 g L^−1^), 0.55 mL of glucose (1 mol L^−1^), and 0.5 mL of sulfured compound stock solution. After inoculation with 1 mL of overnight *T. maritima* culture, final concentrations of NaHCO_3_, glucose, and sulfured compound in the culture medium were 2 g L^−1^, 25, and 0.3 mmol L^−1^ sulfur equivalent, respectively. In this study, two successive cultures, incubated at 80 °C, were done in triplicate for each sulfured compound.

### Culture media for the bioreactor experiments

The basal medium BM for the bioreactor contained (per liter): NaCl 20 g, Yeast extract (Fluka Biochemical, Spain) 1 g, NH_4_Cl 0.5 g, KH_2_PO_4_ 0.3 g, K_2_HPO_4_ 0.3 g, MgCl_2_ 0.2 g, KCl 0.1 g, CaCl_2_ 0.1 g, and Balch trace mineral element solution 10 mL [58]. The BM medium was supplemented with glucose (25 or 60 mmol L^−1^) and thiosulfate at various concentrations ranging between 0 and 0.24 mmol L^−1^. The medium was adjusted to pH 6.5 with 1 mol L^−1^ KOH. Fifteen liters of medium were prepared routinely in a tank (20 L), autoclaved at 120 °C for 45 min, and then cooled down to room temperature under an O_2_-free-N_2_ gas stream. The medium tank was then connected, under sterile conditions, to the feed pump of the bioreactor, and maintained continuously under a stream of O_2_-free N_2_.

### Experimental material and bioreactor

*Thermotoga maritima* was batch cultured in a 2.3 L double-jacket glass bioreactor (FairMenTec, France) with a 1.5 L working volume. The fermentor was run with stirring driven by two axial impellers, and was equipped with sensors to monitor temperature (Prosensor pt 100, France), pH (Mettler Toledo InPro 3253, Switzerland), and redox potential (Mettler Toledo InPro 3253, Switzerland). The incoming gas stream (O_2_-free N_2_ or O_2_-free N_2_ and H_2_), prepared via one or two mass-flow meters (Bronkhorst, range 0–100 or 0–100 and 0–10 mL min^−1^, Netherland, respectively), was injected through a nozzle immersed in the bioreactor. The steam in the outgoing gas stream was condensed in a water-cooler glass exhaust [temperature controlled at 4 °C with a cooling bath equipped with a pump (Julabo SE 6, France)] to prevent liquid loss in the bioreactor (water–vapor condensates were returned to the culture vessel). On the outgoing gas streamline, downstream from the water-cooler glass exhaust, a micro-GC, equipped with a catharometric detector (MS5A, SRA Instrument, France), a GC-FPD, equipped with a flame photometric detector (PR 2100, Perichrom, France), and a CARBOCAP CO_2_ probe (Vaisala GMT 221, Finland), allowed online measurement of H_2_, N_2_ (micro-GC), H_2_S (GC-FPD), and CO_2_ (Probe) contents (see below for the analytic conditions). To prevent air from entering the bioreactor, the outgoing gas streamline was closed off by a hydraulic seal (2 cm deep immersion in oil). The bioreactor was heated by hot-water circulation in the double jacket using a heated bath equipped with a pump (Julabo F25, France). Bioreactor liquid volume and NaOH consumption, which was used to regulate culture pH, were followed via two scales [Sartorius Combics 1 and BP 4100 (France), respectively]. Temperature, pH, gas stream flow rates, and stirrer speed were regulated through control units (local loops). The bioreactor was connected to two pumps dedicated to the supply of fresh culture medium and to empty the reactor. All this equipment was connected to a Wago PLC (France) via a serial link (RS232/RS485), a 4–20 mA analog loop or a digital signal. The PLC was connected to a computer for process monitoring and data acquisition. BatchPro software (Decobecq Automatismes, France) was used to monitor and manage the process with good flexibility and total traceability.

### Operating conditions for the bioreactor

Before each series of fermentation cycles, the reactor was dismantled, washed, and sterilized by autoclaving at 120 °C for 30 min. One series comprised about 15 successive fermentation cycles. One fermentation cycle included three steps: the reactor feeding, the fermentation phase, and the reactor emptying.

For each experimental condition tested, three or four successive fermentation cycles were carried out. In general, the two or three last ones were reproducible for growth and fermentation patterns.

Description of the three steps of a fermentation cycle:

Step 1: Reactor feeding. The reactor was filled with 1.4 L of fresh basal medium BM supplemented with glucose and thiosulfate depending on the experiments. During the filling step, an incoming gas stream (O_2_-free N_2_) adjusted at 500 mL min^−1^ was used to maintain the anoxia in the bioreactor.

Step 2: Fermentation phase. For the first fermentation cycle of a series, the bioreactor inoculation was performed with 100 mL of a recent *T. maritima* culture coming from serum bottles. For the next fermentation cycles, 100 mL of the previous fermentation (n − 1) was kept in the bioreactor to inoculate the current fermentation cycle (n).

For the fermentation phase, temperature and stirrer speed were regulated at 80 ± 0.5 °C and 350 ± 5 rpm, respectively. pH was regulated at 7.0 ± 0.1 by adding 1 mol L^−1^ NaOH. pH and redox probes were calibrated separately at 80 °C with pH −4.22 and −7.04 buffers (Mettler Toledo, Switzerland) and a 124 mV redox buffer at pH 7.0 (Mettler Toledo, Switzerland), respectively. The probe calibrations were verified after each series of fermentation cycles (about every 15 fermentation cycles).

At the beginning of the fermentation phase (step 2), the incoming gas stream (O_2_-free N_2_) was adjusted initially at 10 mL min^−1^ and maintained until the hydrogen percentage in the bioreactor outgoing gas reached the set point of 5%. When the set point was reached, the fermentation process controlled the debit of the incoming gas stream (O_2_-free N_2_) to maintain the hydrogen into the outgoing gas at 5% until the end of the fermentation phase.

Regarding the preliminary experiments, focusing on the effects of partial pressure of hydrogen on growth and glucose catabolism in *T. maritima*, different incoming gaseous mixtures (H_2_/N_2_) (v/v), in a range of (85/15) to (1/99), were used to perfuse the culture. For these experiments, the debit of the incoming gaseous mixtures was constant and adjusted at 50 mL min^−1^ during the fermentation phase.

Step 3: Reactor emptying. The end of the fermentation phase (step 2) was characterized by a decrease of the regulated debit of the incoming gas stream (O_2_-free N_2_) due to the hydrogen production weakening by glucose starvation. Thus, when this debit fell below 15 mL min^−1^, the process triggered the emptying phase of the bioreactor, consisting in removing 1.4 L of culture from the bioreactor, leaving 100 mL to be used as inoculum for the next fermentation cycle. The process was therefore ready for a new cycle.

### Analytical methods

All growths of *T. maritima* were followed by measuring optical density (OD). OD was determined in triplicate at 600 nm with a S2100 Diode array UV–Visible spectrophotometer (WPA Biowave, France). Cell dry weight was determined as one unit OD corresponding to 330 mg L^−1^.

As described earlier, the gas produced during fermentation runs were analyzed continuously with a micro-GC, a GC-FPD, and a CO_2_ probe. Regarding the micro-GC, dedicated to H_2_ and N_2_ measurements, the temperatures of the injector, the column, and the detector were adjusted to 90, 100, and 100 °C, respectively. The pressure of argon, used as carrier gas, was 200 kPa. The gas analysis was repeated every 2 min. The chromatogram treatments were performed by SOPRANE software (SRA Instrument, France).

Regarding the GC-FPD, dedicated to the measure of the H_2_S present in the out-coming gas, the column was a capillary RESEK RTX-1 and the detector was a flame photometer. Operating conditions were as follows: the gradient for oven-temperature increase was adjusted from 50 to 200 °C with a rate of 15 °C per minute, the temperatures of injector and detector were adjusted to 180 and 230 °C, respectively. The pressure of helium, used as carrier gas, was 60 kPa. The frequency of sample-injection gas was set every 20 min and the chromatogram treatments were performed via the WINILAB III software (Perichrom, France).

Glucose, acetate, lactate, and fructose concentrations were determined by HPLC as follows: 1 mL of culture sample was centrifuged for 5 min at 14500 rpm, and 20 L was then loaded onto an Animex HPX-87H column (Biorad) set at 35 °C, and eluted at 0.5 mL min^−1^ with a H_2_SO_4_ solution (0.75 mmol L^−1^). The product concentrations were determined with a differential refractometer detector (Shimadzu RID 6 A, Japan) connected to a computer running WINILAB III software (Perichrom, France). All analyses were performed in triplicate. l-alanine concentrations in centrifuged culture samples were determined by HPLC as described by Moore et al. [60]. Microbial extracellular polysaccharides (EPS) were quantified in centrifuged culture sample by the colorimetric method described by Dubois et al. [61]. Throughout this paper, EPS values were converted in glucose equivalent and expressed in mmol L^−1^.

### Determination of H_2_ and CO_2_ production rates

During the experiments, the data of N_2_ debits and the gas analyses (N_2_, H_2_, and CO_2_) were recorded and used to calculate the fluxes of CO_2_ and H_2_, which then led to the cumulative amounts of CO_2_ and H_2_ produced in the bioreactor.

To determine the production of H_2_ and CO_2_, we used a mathematical model based on the material balances of the 3 gaseous compounds (N_2_, H_2_, and CO_2_):

1 $$\frac{{{\text{d}}p_{{{\text{N}}_{2} }}^{\text{out}} }}{{{\text{d}}t}} = \frac{{Q_{{{\text{N}}_{2} }} }}{{\left( {V_{\text{HR}} - V_{\text{Steam}} } \right)}} \times p_{{{\text{N}}_{2} }} - \frac{{Q_{T}^{\text{out}} }}{{\left( {V_{\text{HR}} - V_{\text{Steam}} } \right)}} \times p_{{{\text{N}}_{2} }}^{\text{out}}$$

2 $$\frac{{{\text{d}}p_{{{\text{CO}}_{2} }}^{\text{out}} }}{{{\text{d}}t}} = \frac{{Q_{{{\text{CO}}_{2} }} }}{{\left( {V_{\text{HR}} - V_{\text{Steam}} } \right)}} \times p_{{{\text{CO}}_{2} }} - \frac{{Q_{T}^{\text{out}} }}{{\left( {V_{\text{HR}} - V_{\text{Steam}} } \right)}} \times p_{{{\text{CO}}_{2} }}^{\text{out}}$$

3 $$\frac{{{\text{d}}p_{{{\text{H}}_{2} }}^{\text{out}} }}{{{\text{d}}t}} = \frac{{Q_{{{\text{H}}_{ 2} }} }}{{\left( {V_{\text{HR}} - V_{\text{Steam}} } \right)}} \times p_{H2} - \frac{{Q_{T}^{\text{out}} }}{{\left( {V_{\text{HR}} - V_{\text{Steam}} } \right)}} \times p_{{{\text{H}}_{2} }}^{\text{out}}$$

4 $$Q_{T}^{\text{out}} \, = \,Q_{{{\text{N}}_{2} }} \, + \,Q\, + \,Q_{{{\text{H}}_{ 2} }}$$

5 $$p_{{{\text{N}}_{2} }}^{\text{out}} \, + \,p_{{{\text{CO}}_{2} }}^{\text{out}} \, + \,p_{{{\text{H}}_{2} }}^{\text{out}} = 100\% {\text{ or 1 bar }}\left( {\text{atmospheric pressure}} \right)$$

Here $p_{{{\text{N}}_{2} }}^{\text{out}}$, $p_{{{\text{CO}}_{2} }}^{\text{out}},$ and $p_{{{\text{H}}_{2} }}^{\text{out}}$ are the partial pressures, in the outlet-gas stream, of N_2_, CO_2_, and H_2_, respectively, $p_{{{\text{N}}_{2} }} \; = \;p_{{{\text{CO}}_{2} }} \; = \;p_{{{\text{H}}_{2} }} \; = \;\;100\%$ are the partial pressures of N_2_ (carrier gas), CO_2_, and H_2_ (biological gas produced during fermentation), respectively, $V_{\text{HR}}$ (960 mL) is the bioreactor headspace volume, and $V_{\text{Steam}}$(320 mL) is water–vapor volume. Vapor volume was calculated according to the Antoine equation at 69 °C (median headspace temperature during the fermentation run). $Q_{{{\text{N}}_{2} }}$, $Q_{{{\text{CO}}_{2} }}$, $Q{}_{{{\text{H}}_{ 2} }},$ and $Q_{T}^{\text{out}}$ are the N_2_, CO_2_, and H_2_ flows and the sum of these three gases, respectively. At 69 °C (headspace temperature), carrier gas flow rate $Q_{{{\text{N}}_{2} }}$ was calculated as follows:

$${\text{Data (mL min}}^{{-1}} ) {\text{ from N}}_{ 2} - {\text{mass}} - {\text{flow meter}} \times \left( {\frac{{273 + 69\;^\circ {\text{C}}}}{{273 + 20\;^\circ {\text{C}}}}} \right)$$.

To determine the total production of CO_2_, ([CO_2_]_aq_ + $[{\text{HCO}}_{3}^{ - } ]$ + $[{\text{CO}}_{3}^{2 - } ]$) in the liquid phase of the bioreactor was estimated by the following relations:

$$[{\text{CO}}_{2} ]_{\text{aq}} \;({\text{mL}}^{ - 1} ) = K_{0} \times p{\text{CO}}_{2} \;({\text{bar}})\quad K_{0} = 0.0127\;{\text{at}}\,\;80\;^\circ {\text{C}}$$

$$[{\text{HCO}}_{3}^{ - } ] \times \;[{\text{H}}^{ + } ] = K_{1} \times [{\text{CO}}_{2} ]_{\text{aq}} \quad K_{1} = 4.93 \times 10^{ - 7} \;{\text{at}}\,\;80\;^\circ {\text{C}}$$

$$[{\text{CO}}_{3}^{2 - } ] \times \;[{\text{H}}^{ + } ] = K_{2} \times [{\text{HCO}}_{3}^{ - } ]\quad K_{2} = 8.18 \times 10^{ - 11} \;{\text{at}}\,\;80\;^\circ {\text{C}}$$

$$[{\text{H}}^{ + } ] = 10^{{ - {\text{pH}}}}$$

Here *p*CO_*2*_ is the partial pressure of CO_2_ in the headspace of the bioreactor.

## Results and discussion

**Fig. 1:**
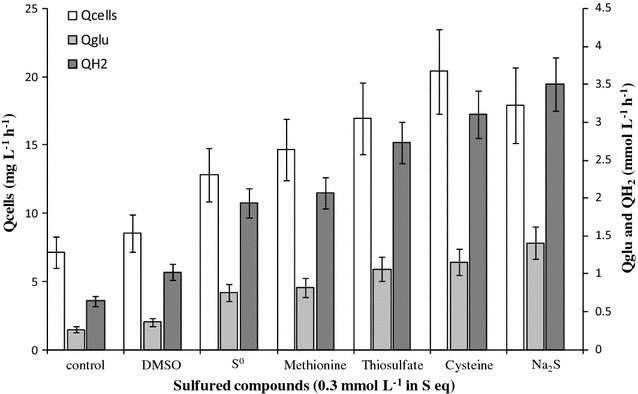
Comparisons of different sulfured nutriments on *Thermotoga maritima* cultures. All sulfured compounds were added at the rate of 0.3 mmol L^−1^ sulfur equivalent in a medium containing glucose (25 mmol L^−1^), yeast extract (1 g L^−1^), and salts (see “Methods” section). DMSO, S°, and Na_2_S meant dimethyl sulfoxide, elementary sulfur, and sodium sulfide, respectively. Productivities of cells (Qcells), and of hydrogen (QH_2_), and consumption of glucose (Qglu) were calculated during the growth phase (after 14.5 h of incubation). All batch cultures were performed in triplicate in serum bottles. The control corresponded to *T. maritima* grown without adding the sulfured compounds

**Fig. 2:**
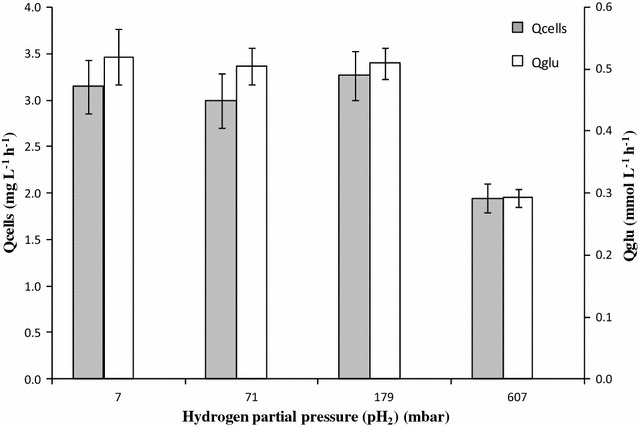
Cellular and hydrogen productivities versus hydrogen partial pressure. Productivities of *T. maritima* cells (Qcells) were expressed in mg per liter of medium and per hour. Glucose consumption rates (Qglu) were expressed in mmol per liter of medium and per hour. These fermentation parameters were calculated during the growth phase from fermentation runs performed in bioreactor (in triplicate). For all fermentations, culture medium contained glucose (60 mmol L^−1^) and yeast extract (1 g L^−1^)

Before carrying out our study on the sulfur-compound effects on *T. maritima* growth, a specific formulation for the growth medium was determined. In order to emphasize the impact of these compounds, the glucose and yeast extract concentrations were determined in such a way that the bacterial growth was limited only by the nutrients present in the yeast extract. This point was essential because of the presence, in the yeast extract, of sulfur compounds such as cystine and methionine. Based on the experiments performed in the bioreactor (see Figs. 1 and 2 presented in the part II) [62], it was established that, in the presence of a glucose excess (concentrations greater than 20 mmol L^−1^), *T. maritima* growth was only limited by the yeast extract for concentrations ranging from 0 to 1 g L^−1^. Consequently, we have chosen, for all the following experiments, to use a culture medium containing 1 g L^−1^ of yeast extract with 25 and 60 mmol L^−1^ glucose for fermentation runs performed in serum bottles and bioreactor, respectively.

### Effects of several sulfur compounds on *T. maritima* growth performed in serum bottles

Dimethyl sulfoxide (DMSO), methionine, cysteine, thiosulfate, elementary sulfur, and sodium sulfide (Na_2_S), at a concentration of 0.3 mmol L^−1^ equivalent sulfur, were tested as sulfured sources on *T. maritima* cultures. These cultures were performed in serum bottles under anoxic conditions in the presence of glucose as energy source.

For all the cultures, results of C-recovery ranged from 88.1 to 96.2%, showing that almost all of the carbon of the fermented glucose was recovered as lactate, acetate, and CO_2_, the latter being estimated by considering that one mole of CO_2_ was produced per mole of acetate (Table 1). In addition, the levels of the molar ratios “H_2_/acetate,” found to be close to 2.0 (between 1.7 and 2.2) (Table 1), indicated that the hydrogen and acetate produced were correctly measured.

**Table 1:** Comparison of *Thermotoga maritima* growths cultivated in serum bottles in the presence of different sulfured nutriments

	Time	Cells	Glu cons	Lactate	Acetate	H_2_	H_2_/acet	C-recovery	Qcells	Qglu	QH_2_
	h	mg L^−1^	mmol L^−1^				mol mol^−1^	%	mg L^−1^ h^−1^	mmol L^−1^ h^−1^	
Control	0	4.8 ± 0.1	0.0	0.0	0.0	0.0	–	–	–	–	–
	14.5	108.2 ± 10.8	3.8 ± 0.2	0.5 ± 0.1	5.4 ± 0.4	9.3 ± 0.9	1.71 ± 0.29	96.2 ± 12.7	7.14 ± 0.75	0.26 ± 0.01	0.64 ± 0.06
	22.0	158.2 ± 15.8	7.1 ± 0.4	0.8 ± 0.1	10.1 ± 0.8	21.3 ± 2.1	2.10 ± 0.35	91.4 ± 12.1	6.98 ± 0.72	0.32 ± 0.02	0.97 ± 0.10
DMSO	0	6.0 ± 0.1	0.0	0.0	0.0	0.0	–	–	–	–	–
	14.5	129.7 ± 13.0	5.2 ± 0.3	0.4 ± 0.0	7.4 ± 0.6	14.9 ± 1.5	2.01 ± 0.33	91.0 ± 12.0	8.53 ± 0.90	0.36 ± 0.02	1.02 ± 0.10
	22.0	160.8 ± 16.1	9.2 ± 0.5	0.8 ± 0.1	13.3 ± 1.1	28.7 ± 2.9	2.17 ± 0.36	88.1 ± 11.7	7.03 ± 0.73	0.42 ± 0.02	1.31 ± 0.13
S°	0	5.9 ± 0.1	0.0	0.0	0.0	0.0	–	–	–	–	–
	14.5	191.8 ± 19.2	11.0 ± 0.5	1.3 ± 0.1	16.3 ± 1.3	28.0 ± 2.8	1.72 ± 0.29	91.7 ± 12.1	12.82 ± 1.32	0.76 ± 0.04	1.93 ± 0.19
	22.0	158.2 ± 15.8	16.6 ± 0.8	3.4 ± 0.3	23.8 ± 1.9	46.1 ± 4.6	1.93 ± 0.32	88.3 ± 11.7	6.93 ± 0.72	0.75 ± 0.04	2.09 ± 0.21
Methionine	0	5.1 ± 0.1	0.0	0.0	0.0	0.0	–	–	–	–	–
	14.5	217.7 ± 21.8	11.9 ± 0.6	1.1 ± 0.1	17.3 ± 1.4	30.0 ± 3.0	1.73 ± 0.29	90.2 ± 11.9	14.67 ± 1.50	0.82 ± 0.04	2.07 ± 0.21
	22.0	227.8 ± 22.8	18.3 ± 0.9	3.1 ± 0.3	26.5 ± 2.1	53.3 ± 5.3	2.01 ± 0.34	89.2 ± 11.8	10.13 ± 1.04	0.83 ± 0.04	2.42 ± 0.24
Thiosulfate	0	4.1 ± 0.1	0.0	0.0	0.0	0.0	–	–	–	–	–
	14.5	*250.6* *±* *25.1*	*15.4* *±* *0.8*	3.6 ± 0.4	*22.0* *±* *1.8*	*39.7* *±* *4.0*	1.80 ± 0.30	94.5 ± 12.5	*16.96* *±* *1.73*	*1.06* *±* *0.05*	*2.74* *±* *0.27*
	22.0	157.0 ± 15.7	17.5 ± 0.9	6.3 ± 0.6	24.1 ± 1.9	47.3 ± 4.7	1.97 ± 0.33	93.0 ± 12.3	6.92 ± 0.71	0.79 ± 0.04	2.15 ± 0.21
Cysteine	0	4.7 ± 0.1	0.0	0.0	0.0	0.0	–	–	–	–	–
	14.5	*300.6* *±* *30.1*	*16.8* *±* *0.8*	2.2 ± 0.2	*25.4* *±* *2.0*	*45.0* *±* *4.5*	1.77 ± 0.30	94.5 ± 12.5	*20.41* *±* *2.07*	*1.16* *±* *0.06*	*3.10* *±* *0.31*
	22.0	158.2 ± 15.8	20.4 ± 1.0	4.1 ± 0.4	30.5 ± 2.4	58.5 ± 5.8	1.92 ± 0.32	90.0 ± 11.9	6.98 ± 0.72	0.93 ± 0.05	2.66 ± 0.27
Na_2_S	0	4.7 ± 0.1	0.0	0.0	0.0	0.0	–	–	–	–	–
	14.5	*264.6* *±* *26.5*	*20.4* *±* *1.0*	4.7 ± 0.5	*28.1* *±* *2.2*	*50.9* *±* *5.1*	1.81 ± 0.3	89.3 ± 11.8	*17.92* *±* *1.82*	*1.41* *±* *0.07*	*3.51* *±* *0.35*
	22.0	234.2 ± 23.4	20.4 ± 1.0	4.7 ± 0.5	30.7 ± 2.5	54.9 ± 5.5	1.79 ± 0.3	94.4 ± 12.5	10.43 ± 1.06	0.93 ± 0.05	2.49 ± 0.25

All sulfured compounds were added at the rate of 0.3 mmol L^−1^ sulfur equivalent in a medium containing glucose (25 mmol L^−1^), yeast extract (1 g L^−1^), and salts (see “Methods” section). DMSO, S°, and Na_2_S meant Dimethyl Sulfoxyde, elementary sulfur, and sodium sulfide, respectively. “glu cons” was glucose consumed during fermentation. Carbon recovery was calculated by taking into account the carbon moles of products (cells, lactate, acetate, and CO_2_) and substrate (glucose). C-cells represented 50% (p/p) of the cells dry weight. CO_2_ was estimated by considering 1 mol of CO_2_ produced per mole of acetate. Qglu was the volume rates of glucose consumption expressed in mg cdw per hour and per liter of culture medium. Qcells and QH_2_ were the productivity of cells and hydrogen, respectively. These productivities were expressed in millimoles cdw per hour and per liter of culture medium. All batch cultures were performed in triplicate in serum bottles

To evaluate the effect of the various sulfur compounds on growth and fermentation of glucose, three parameters, cellular production rates (Qcells), glucose consumption rates (Qglu), and hydrogen production rates (QH_2_), calculated during the first 14.5 h of fermentation (growth phase), were used (Fig. 1).

The results in Fig. 1 showed that, by comparison with the control culture (culture grown without adding the sulfured compounds) the presence of DMSO had no significant effect on *T. maritima* growth and glucose fermentation. In contrast, the presence of the five other sulfur compounds—elementary sulfur, methionine, thiosulfate, cysteine, and sodium sulfide—accelerated *T. maritima* growth and fermentation, as indicated by the increasing of Qcells, Qglu, and QH_2_. Finally, among these compounds, thiosulfate, cysteine, and sodium sulfide were significantly the most efficient (Fig. 1).

Childers et al. [51] already reported beneficial effects of sulfur compounds such as cystine, dimethylsulfide, or elementary sulfur on *T. neapolitana* growth, a species close to *T. maritima* [51]. Similarly, it was reported that most *Thermotoga* species, including *T. maritima,* were able to reduce thiosulfate and/or elementary sulfur into H_2_S and that their growths were enhanced in the presence of thiosulfate and/or elementary sulfur [32, 35, 42, 47, 48, 50]. So far, it is generally admitted that the improving of *Thermotoga* species growth, due to the reductive process of sulfur compounds (elementary sulfur, thiosulfate, cystine, dimethyl sulfide, or polysulfur) into sulfide, was the result of a detoxifying process preventing H_2_ accumulation, a powerful inhibitor for growth, rather than an energy-yielding electron-sink reaction [32, 35, 39, 47]. However, in our experimental conditions, the idea of a “detoxifying process preventing H_2_ accumulation” cannot be retained to explain the observed enhancing of *T. maritima* growth since the concentration of thiosulfate (0.15 mmol L^−1^) was too low. Indeed, if the added thiosulfate was only used for oxidizing H_2_, 0.15 mmol L^−1^ of thiosulfate could only allow oxidizing 0.6 mmol L^−1^ of H_2_ (4 mol of H_2_ are necessary to reduce 1 mol of thiosulfate into sulfide). This amount of oxidized hydrogen (0.6 mmol L^−1^) appears insignificant in comparison to the 47.3 mmol L^−1^ of hydrogen produced during the fermentation of glucose (Table 1). In consequence, given the small amount of hydrogen potentially oxidized by the thiosulfate, the enhancing of *T. maritima* growth cannot be attributed to a detoxification concept. The only remaining valid assumption would be that thiosulfate should be considered, under our experimental conditions, as a sulfur source dedicated to the anabolism and thus to the synthesis of cellular components such as proteins.

### Experiments performed in bioreactor

To deepen and specify the stimulating effect of sulfur-compound low concentrations on the fermentation of glucose by *T. maritima*, thiosulfate was selected as the sulfur source for all subsequent experiments in the bioreactor. In contrast to Na_2_S and cysteine, thiosulfate is both non-volatile and thermally stable under our experimental conditions (temperature and pH were controlled at 80 °C and 7.0, respectively). In addition, to minimize as much as possible, the inhibitory effects of hydrogen on growth and metabolism of *T. maritima*, the reactor was equipped with a specific device to control the hydrogen partial pressure in the headspace (see “Methods” section).

#### Determination of the critical limit of hydrogen partial pressures (pH_2_) to prevent the inhibition of *T. maritima* cultures

In order to determine the technical specifications of the pH_2_ control, enabling, at the same time, on one hand, not to inhibit the *T. maritima* cultures, and, on the other hand, to estimate correctly the hydrogen and CO_2_ productions, several fermentation runs were performed varying the pH_2_, in the bioreactor headspace, within a range from 7 to 607 mbar at 80 °C. To perform these experiments, the bioreactor was fed with different incoming gaseous mixtures (H_2_/N_2_) used at a constant total debit (50 mL min^−1^).

The results presented in Fig. 2 showed that the cellular production rates (Qcells) and the glucose consumptions rates (Qglu) were unaffected when pH_2_ was maintained in a range of 7.1–178.5 mbar. In contrast, the providing of H_2_ at a partial pressure of 607 mbar within the bioreactor headspace caused an approximate twofold decrease in Qglu and Qcells levels (Fig. 2).

These results were different from those reported by Schroder et al. [39], which have shown that *T. maritima* growth was reduced even for hydrogen partial pressures as low as 28 mbar. On the contrary, our results were totally consistent with numerous authors who concluded that (1) a pH_2_ lower than 200 mbar is required for a fully functional hydrogen-producing reactor in which the growth was optimal [44, 63–65], and (2) a pH_2_ estimated at 2900 mbar at 85 °C was completely inhibiting *T. maritima* growth [32].

Furthermore, as already mentioned in literature [39, 42], our results showed that the hydrogen partial pressure increase, within the bioreactor, led to a shift of the glucose catabolism from acetate towards lactate (molar ratios lactate/glucose increased from 0.5 to 0.8 and molar ratios acetate/glucose changed inversely from 1.3 to 1.0 at 7 and 607 mbar of pH_2_, respectively) (Table 2).

**Table 2:** *Thermotoga maritima* cultures performed in bioreactor in the presence of different hydrogen partial pressures (pH_2_)

pH_2_	Time		Cells	Glu cons	Lactate	Acetate	H_2_	CO_2_	Acet/glu	Lac/glu	Eh	C-recovery
mbars	h		mg L^−1^	mmol L^−1^					mol mol^−1^		mV	%
7.1 ± 0.4	*t*_0_	0.0	14.8 ± 2.0	0.0	0.0	0.0	0.0	0.0	–	–	−130 ± 5	–
	*t*_1_	6.7	17.5 ± 3.2	0.3 ± 0.3	0.1 ± 0.1	0.6 ± 0.3	0.4 ± 0.0	0.4 ± 0.1	–	–	−135 ± 6	–
	*t*_2_	41.7	127.5 ± 9.7	18.5 ± 1.0	8.9 ± 1.0	23.6 ± 1.2	44.5 ± 3.3	22.5 ± 1.8	1.3 ± 0.1	0.5 ± 0.1	−314 ± 14	91.2 ± 15.1
	*t*_3_	49.8	122.5 ± 9.7	19.8 ± 1.1	10.5 ± 0.5	25.0 ± 1.4	46.4 ± 3.6	23.3 ± 1.9	1.3 ± 0.1	0.5 ± 0.0	−279 ± 30	92.1 ± 15.3
71.4 ± 2.1	*t*_0_	0.0	14.7 ± 2.8	0.0	0.0	0.0	0.0	–	–	–	−152 ± 8	–
	*t*_1_	5.5	19.1 ± 2.7	0.5 ± 0.2	0.2 ± 0.1	0.7 ± 0.2	1.4 ± 0.6	0.3 ± 0.0	–	–	−151 ± 14	–
	*t*_2_	40.4	123.6 ± 9.6	18.1 ± 1.2	8.8 ± 0.6	22.5 ± 2.1	43.0 ± 4.4	20.0 ± 2.0	1.2 ± 0.1	0.5 ± 0.0	−409 ± 20	88.4 ± 14.7
	*t*_3_	46.2	118.2 ± 9.3	19.7 ± 1.4	11.0 ± 0.6	24.6 ± 2.4	48.0 ± 5.0	21.8 ± 2.2	1.2 ± 0.1	0.6 ± 0.0	−372 ± 19	91.6 ± 15.2
178.5 ± 3.5	*t*_0_	0.0	12.7 ± 1.0	0.0	0.0	0.0	0.0	–	–	–	−161 ± 4	–
	*t*_1_	5.5	22.7 ± 1.7	0.2 ± 0.0	0.1 ± 0.1	0.2 ± 0.0	0.6 ± 0.0	0.3 ± 0.0	–	–	−176 ± 4	–
	t_2_	38.2	129.8 ± 9.7	16.9 ± 0.8	9.2 ± 0.4	19.9 ± 1.0	39.3 ± 2.9	19.2 ± 1.5	1.2 ± 0.1	0.5 ± 0.0	−443 ± 11	90.1 ± 15.0
	*t*_3_	39.0	127.3 ± 9.6	17.2 ± 0.9	9.4 ± 0.5	20.1 ± 1.0	40.0 ± 3.0	19.5 ± 1.6	1.2 ± 0.1	0.5 ± 0.0	−426 ± 11	89.8 ± 14.9
606.9 ± 18.7	*t*_0_	0.0	18.9 ± 1.4	0.0	0.0	0.0	*a*	–	–	–	−239 ± 6	–
	*t*_1_	4.4	23.9 ± 1.8	1.0 ± 0.1	0.0 ± 0.0	0.0 ± 0.0	*a*	0.3 ± 0.0	–	–	−240 ± 6	–
	*t*_2_	41.7	96.4 ± 7.2	11.9 ± 0.6	8.9 ± 0.4	12.0 ± 0.6	*a*	11.1 ± 0.9	1.0 ± 0.1	0.7 ± 0.1	−449 ± 11	90.6 ± 15.0
	*t*_3_	48.5	96.3 ± 7.2	13.4 ± 0.7	11.0 ± 0.6	13.0 ± 0.7	*a*	12.2 ± 1.0	1.0 ± 0.1	0.8 ± 0.1	−440 ± 11	92.9 ± 15.4

The culture medium contained initially 25 mmol L^−1^ of glucose and 1 g L^−1^ of yeast extract. Operating conditions for the regulations of pH, agitation, and temperature were adjusted to 7.0, 350 rpm, and 80 °C, respectively. The pH_2_, as reported in the table, were partial pressures of hydrogen maintained in the headspace of the bioreactor. 7.1, 71.4, 178.5, and 606.9 mbar were obtained with (H_2_/N_2_) gas mixtures: (1/99), (10/90), (25/75), and (85/15), respectively, and injected through the bioreactor at a constant total debit of 50 mL min^−1^ under a pressure close to 1 bar. Times t_1_ to t_2_, and t_3_ corresponded to the growth phases and to the end of the fermentation run, respectively

*a* indicated that, under these experimental conditions [gas mixtures (H_2_/N_2_):(85/15)], biological productions of hydrogen could not be determined with sufficient precision

*Eh* corresponds to the measurement, within the bioreactor, of the reduction potential relative to a standard hydrogen electrode. Carbon recovery was calculated as in the Table 1. All the batch cultures were performed in triplicate in bioreactor

Moreover, it is noteworthy that, whatever the pH_2_ tested, the fermentation of glucose by *T. maritima* led to a significant reduction of the culture medium as indicated by the decrease of Eh measurements during the growth phases (between *t*_1_ and *t*_2_) (Table 2). In addition, the results showed that the initial Eh measurements (at *t*_0_ before starting the fermentation phase) were conversely correlated to the level of pH_2_ (Eh measurements at t_0_ decreased from −130 to −239 mV when pH_2_ increased from 7 to 607 mbar, respectively) (Table 2). Taken together, these results indicated that the phenomenon of reduction of the culture medium was due to both biological and chemical activities. As discussed in a previous study [43], this capacity of *T. maritima* to reduce the culture medium by itself suggested that the reducing compounds such as cysteine and/or Na_2_S usually added in anaerobic medium cultures for Thermotogales growth were unnecessary.

In addition, concerning the estimates of H_2_ and CO_2_ productions, the monitoring of the fermentation runs, carried out with an incoming gaseous mixture (N_2_/H_2_) containing 85% of hydrogen (pH_2_ at 607 mbar), has revealed that, under these conditions, the hydrogen production accuracy was insufficient. In contrast, for the other fermentation runs, fed with gaseous mixtures containing less than 25% of H_2_ (batches with pH_2_ lower than 179 mbar), both measures and method to calculate H_2_ and CO_2_ productions (“Determination of H_2_ and CO_2_ production rates” in the “Methods” section) were correct as confirmed by molar ratios H_2_/CO_2_ and CO_2_/acetate close to 2 and 1, respectively (corresponding to *T. maritima* glucose catabolism [39]) (Table 2).

In accordance with our results, we have therefore chosen, for all the following fermentation runs, to control pH_2_ at a maximal value of 35 mbar, corresponding to a maximum of 5% (v/v) H_2_ in the gaseous outflow of the bioreactor (for more details on the pH_2_ control see paragraph ≪Operating conditions for the bioreactor≫ in the “Methods” section). Under these experimental conditions, our results showed that hydrogen and CO_2_ productions were correctly estimated, and growth and glucose catabolism were unaffected by H_2_.

#### *Thermotoga maritima* growth in culture medium limited by thiosulfate

The effects of the limitation of thiosulfate, as the main sulfur growth nutriment, were studied on the growth and on the pattern of glucose fermentation products in *T. maritima*. Fermentation runs were performed varying the thiosulfate concentration within a range from 0 to 1.0 mmol L^−1^, in the bioreactor, with a culture medium containing yeast extract and high concentrations of glucose (1 g L^−1^ and 60 mmol L^−1^, respectively).

**Fig. 3:**
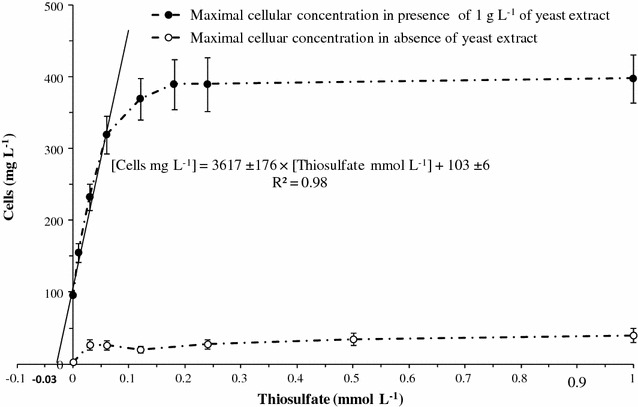
Maximum cellular concentrations versus thiosulfate concentrations. In the absence of yeast extract, maximal biomass is obtained with glucose (25 mmol L^−1^) as energy source. The cultures were performed in triplicate in serum bottles after nine subcultures under the same conditions. In the presence of yeast extract at 1 g L^−1^, maximal biomass is obtained with glucose (60 mmol L^−1^). These cultures were performed in triplicate in bioreactor

Under our experimental conditions, the thiosulfate concentration had a significant effect on the maximal cellular concentration obtained during fermentation runs (“cells” values reported at *t*_2_ in Table 3). Figure 3 shows that the cellular growth was limited by thiosulfate only when its concentration was lower than 0.06 mmol L^−1^. For higher concentrations up to 1.0 mmol L^−1^, cellular concentration approached an upper limit of 400 mg L^−1^, probably revealing other nutritional limitations, or back-inhibition by fermentation products such as acetate and lactate. In thiosulfate-limited batch cultures (concentrations ranging from 0 to 0.06 mmol L^−1^), the cellular yield on thiosulfate (*Y X/Thio*) could be determined at 3617 ± 176 mg (cell dry weight) of cells per mmol of thiosulfate initially present in the culture medium (Fig. 3). Otherwise, the culture, performed in mineral medium (absence of yeast extract) with glucose as sole carbon and energy source, showed that thiosulfate was essential for *T. maritima* growth (Fig. 3). Under these culture conditions, the cellular concentrations reached 27 mg L^−1^ with 0.03 mmol L^−1^ of thiosulfate, up to a limit of approximately 40 mg L^−1^ with 1 mmol L^−1^ (Fig. 3). The cellular productions, obtained with 1 g L^−1^ of yeast extract (about 128 mg L^−1^ of cells as maximum concentration) (Table 3) without adding thiosulfate, could be explained by the sulfured compounds, such as cystine and methionine, present in the yeast extract. In addition, it could be concluded that among all the nutrients provided by the yeast extract, the sulfur compounds were likely those limiting *T. maritima* growth. Finally, the extrapolation of the linear regression presented in Fig. 3 suggested that the sulfur nutrients present in 1 g L^−1^ of yeast extract were equivalent at 0.03 mmol L^− 1^ of thiosulfate in terms of effect on the growth of *T. maritima*. On the other hand, as shown in Fig. 3, the significant offset between the cellular concentrations obtained in the presence and absence of yeast extract, with thiosulfate in excess (concentrations greater than 0.2 mmol L^−1^), unambiguously indicated that the yeast extract also provided, in addition to sulfured compounds, some specific nutritional factors strongly stimulating *T. maritima* growth. These compounds could be some vitamins as reported by Childers et al. [51] rather than amino acids, demonstrated to be poorly used by *T. maritima* [46].

**Table 3:** *Thermotoga* grown in the presence of different concentrations of thiosulfate

Thiosulfate	Time		Cells	Glu cons	Lactate	Acetate	CO_2_	H_2_	C^1^-recovery	l-Alanine	EPS	C^2^-recovery
m mol	h		mg L^−1^	m mol					%	mmol		%
0.0	*t*_0_	0.0	19.3 ± 2.2	0.0	0.0	0.0	0.0	0.0	–	0.0	0.0	–
	*t*_1_	4.8	31.8 ± 2.5	1.3 ± 0.3	0.2 ± 0.0	0.5 ± 0.2	0.2 ± 0.1	0.3 ± 0.0	–	–	–	–
	*t*_2_	29.5	127.6 ± 9.7	16.4 ± 1.1	4.9 ± 0.5	11.8 ± 0.8	11.1 ± 1.0	23.2 ± 2.0	54.9 ± 12.1	1.39 ± 0.2	4.7 ± 1.2	87.2 ± 18.1
	*t*_3_	30.6	119.8 ± 10.9	17.7 ± 1.9	5.4 ± 0.6	12.8 ± 1.0	11.9 ± 1.1	25.0 ± 2.2	54.5 ± 12.0	–	–	–
0.01	*t*_0_	0.0	16.8 ± 3.1	0.0	0.0	0.0	0.0	0.0	–	–	–	–
	*t*_1_	3.1	22.8 ± 1.7	2.1 ± 0.5	0.1 ± 0.0	0.2 ± 0.0	0.2 ± 0.0	0.4 ± 0.0	–	–	–	–
	*t*_2_	22.8	178.0 ± 15.4	19.8 ± 1.1	10.0 ± 1.0	16.0 ± 0.8	15.0 ± 1.2	30.7 ± 2.3	70.4 ± 11.7	–	–	–
	*t*_3_	23.0	177.4 ± 15.2	20.0 ± 1.1	10.2 ± 1.1	16.0 ± 0.8	15.0 ± 1.2	31.0 ± 2.3	70.6 ± 11.7	–	–	–
0.03	*t*_0_	0.0	23.6 ± 3.9	0.0	0.0	0.0	0.0	0.0	–	–	–	–
	*t*_1_	6.7	32.1 ± 3.0	0.0 ± 0.0	0.0 ± 0.0	0.0 ± 0.0	0.2 ± 0.0	0.3 ± 0.0	–	–	–	–
	*t*_2_	24.1	265.0 ± 22.5	25.2 ± 2.3	6.8 ± 1.9	27.5 ± 2.1	27.3 ± 2.6	53.5 ± 4.6	74.6 ± 12.4	–	–	–
	*t*_3_	27.5	264.1 ± 22.4	28.0 ± 1.5	8.2 ± 0.7	30.6 ± 1.9	29.7 ± 2.5	57.9 ± 4.8	74.7 ± 12.4	–	–	–
0.06	t_0_	0.0	26.1 ± 2.8	0.0	0.0	0.0	0.0	0.0	–	–	–	–
	*t*_1_	1.8	33.7 ± 4.0	0.7 ± 0.2	0.0 ± 0.0	0.1 ± 0.0	0.1 ± 0.1	0.3 ± 0.0	–	–	–	–
	*t*_2_	20.4	353.5 ± 26.5	38.2 ± 2.0	18.0 ± 1.8	37.7 ± 2.3	35.7 ± 3.0	73.2 ± 5.9	78.1 ± 13.0	–	–	–
	*t*_3_	22.3	352.3 ± 26.4	38.5 ± 2.0	18.1 ± 1.8	38.2 ± 2.4	35.8 ± 3.0	73.3 ± 5.9	77.9 ± 12.9	–	–	–
0.12	*t*_0_	0.0	24.4 ± 2.3	0.0	0.0	0.0	0.0	0.0	–	0.0	0.0	–
	*t*_1_	3.0	34.1 ± 2.8	1.1 ± 0.3	0.0 ± 0.0	0.8 ± 0.4	0.3 ± 0.1	0.3 ± 0.0	–	–	–	–
	*t*_2_	17.9	404.0 ± 32.3	42.4 ± 2.3	15.9 ± 1.5	47.0 ± 2.5	44.2 ± 3.7	90.5 ± 7.0	79.2 ± 13.2	3.8 ± 0.3	3.6 ± 0.9	92.2 ± 19.0
	*t*_3_	23.2	396.6 ± 32.8	45.7 ± 2.5	15.4 ± 1.6	52.4 ± 3.3	51.9 ± 4.9	99.7 ± 8.3	79.6 ± 13.2	–	–	–
0.18	*t*_0_	0.0	25.3 ± 2.5	0.0	0.0	0.0	0.0	0.0	–	–	–	–
	*t*_1_	1.2	32.7 ± 3.3	0.3 ± 0	0.0 ± 0	0.0 ± 0	0.4 ± 0.1	0.4 ± 0.1	–	–	–	–
	*t*_2_	16.8	428.8 ± 32.2	45.0 ± 2.2	23.3 ± 2.3	44.5 ± 2.5	44.6 ± 4.0	86.7 ± 7.0	81.5 ± 13.5	–	–	–
	*t*_3_	17.0	418.0 ± 33.0	45.4 ± 2.3	23.4 ± 2.3	45.0 ± 2.2	44.7 ± 5.0	86.9 ± 8.2	81.3 ± 13.5	–	–	–
0.24	*t*_0_	0.0	27.5 ± 2.1	0.0	0.0	0.0	0.0	0.0	–	0.0	0.0	–
	*t*_1_	2.1	33.8 ± 2.6	0.9 ± 0.3	0.0 ± 0.0	0.0 ± 0.0	0.2 ± 0.0	0.5 ± 0.0	–	–	–	–
	*t*_2_	17.0	423.9 ± 31.8	41.7 ± 2.1	22.5 ± 1.5	43.4 ± 3.5	40.0 ± 3.2	84.0 ± 7.0	84.3 ± 14.0	3.8 ± 0.2	2.9 ± 0.7	95.8 ± 19.8
	*t*_3_	22.8	422.4 ± 31.7	43.8 ± 2.2	26.4 ± 1.4	46.1 ± 3.3	42.2 ± 3.8	88.6 ± 8.9	87.5 ± 14.5	–	–	–

Thiosulfate	Time		Cells/glu	Acet/glu	Lac/glu	H_2_/glu	l-ala/glu	EPS/glu	q glucose	q H_2_
m mol	h		g mol^−1^		mol mol^−1^				mmol g^−1^ h^−1^	
0.0	*t*_0_	0.0	–	–	–	–	–	–	–	–
	*t*_1_	4.8	–	–	–	–	–	–	–	–
	*t*_2_	29.5	6.3 ± 0.6	0.7 ± 0.1	0.3 ± 0.0	1.4 ± 0.2	0.08 ± 0.01	0.29 ± 0.05	8.9 ± 1.3	13.4 ± 2.3
	*t*_3_	30.6	–	0.7 ± 0.1	0.3 ± 0.0	1.4 ± 0.2	–	–	–	–
0.01	*t*_0_	0.0	–	–	–	–	–	–	–	–
	*t*_1_	3.1	–	–	–	–	–	–	–	–
	*t*_2_	22.8	8.7 ± 0.9	0.8 ± 0.1	0.5 ± 0.0	1.6 ± 0.2	–	–	12.0 ± 1.8	20.4 ± 3.5
	*t*_3_	23.0	–	0.8 ± 0.1	0.5 ± 0.1	1.6 ± 0.2	–	–	–	–
0.03	*t*_0_	0.0	–	–	–	–	–	–	–	–
	*t*_1_	6.7	–	–	–	–	–	–	–	–
	*t*_2_	24.1	9.3 ± 1.4	1.1 ± 0.1	0.3 ± 0.1	2.1 ± 0.2	–	–	13.1 ± 2.0	27.7 ± 4.7
	*t*_3_	27.5	–	1.1 ± 0.1	0.3 ± 0.1	2.1 ± 0.2	–	–	–	–
0.06	*t*_0_	0.0	–	–	–	–	–	–	–	–
	*t*_1_	1.8	–	–	–	–	–	–	–	–
	*t*_2_	20.4	8.5 ± 0.8	1.0 ± 0.1	0.5 ± 0.0	1.9 ± 0.2	–	–	14.8 ± 2.2	28.7 ± 4.9
	*t*_3_	22.3	–	1.0 ± 0.1	0.5 ± 0.0	1.9 ± 0.2	–	–	–	–
0.12	*t*_0_	0.0	–	–	–	–	–	–	–	–
	*t*_1_	3.0	–	–	–	–	–	–	–	–
	*t*_2_	17.9	8.9 ± 0.8	1.1 ± 0.1	0.4 ± 0.0	2.1 ± 0.2	0.09 ± 0.01	0.08 ± 0.01	18.5 ± 2.8	40.4 ± 6.9
	*t*_3_	23.2	–	1.1 ± 0.1	0.3 ± 0.0	2.2 ± 0.2	–	–	–	–
0.18	*t*_0_	0.0	–	–	–	–	–	–	–	–
	*t*_1_	1.2	–	–	–	–	–	–	–	–
	*t*_2_	16.8	8.7 ± 0	1.0 ± 0.1	0.5 ± 0.0	1.9 ± 0.2	–	–	18.8 ± 2.8	36.3 ± 6.2
	*t*_3_	17.0	–	1.0 ± 0.1	0.5 ± 0.0	1.9 ± 0.2	–	–	–	–
0.24	*t*_0_	0.0	–	–	–	–	–	–	–	–
	*t*_1_	2.1	–	–	–	–	–	–	–	–
	*t*_2_	17.0	9.6 ± 0.9	1.0 ± 0.1	0.5 ± 0.0	2.0 ± 0.2	0.09 ± 0.01	0.07 ± 0.01	17.9 ± 2.7	36.6 ± 6.2
	*t*_3_	22.8	–	1.1 ± 0.1	0.6 ± 0.0	2.0 ± 0.2	–	–	–	–

q glucose and q H_2_ were the specific glucose consumption rate and the specific hydrogen productivity, respectively. They were calculated in taking into account the linear increase of cell concentration found during the growth phases (from t_1_ to t_2_). They were expressed in mmol per g of cells (dw) and per hour

In order to evaluate the sulfur quantity from thiosulfate incorporated in the cellular material, the quantities of sulfur present both in the cells and originally in the culture medium were calculated for two specific culture conditions (Table 4). The first condition corresponds to organic sulfur-limited growth provided by 1 g L^−1^ of yeast extract (medium without adding thiosulfate). The second condition corresponds to a growth limited by the sulfur provided by both 0.06 mmol L^−1^ of thiosulfate and 1 g L^−1^ of yeast extract as shown in Fig. 3. The amount of organic sulfur (found in the form of cystine, a cysteine dimer, and methionine) present in 1 g L^−1^ yeast extract was calculated from the yeast extract composition provided by Sigma (0.008 mmol of cystine and 0.054 mmol of methionine or about 0.07 mmol of organic sulfur (S-YE) per gram of yeast extract). Both these two amino acids can be assimilated by *T. maritima* as previously shown (Fig. 1). The amount of sulfur present in 0.06 mmol of thiosulfate represented 0.12 mmol (S-thiosulfate). Finally, cellular sulfur (S-cells) was calculated from the elemental composition of *T. maritima* cells given by Kelly et al. (C1 H1.6 O0.6 N0.2 S0.005) [46] and from the maximum cell concentrations obtained under both culture conditions (Tables 3 and 4).

**Table 4:** Cellular sulfur and sulfured nutriments in two culture conditions in the presence and absence of thiosulfate

Growth condition	S-YE	S-thiosulfate	Cells	S-cells	S-cells/S-(YE + thio)
	mmol L^−1^		mg L^−1^	mmol L^−1^	%
Growth in presence of yeast extract (1 g L^−1^) and in absence of thiosulfate	0.07	0.00	128	0.025	35
Growth in presence of both yeast extract (1 g L^−1^) and thiosulfate (0.06 mmol L^−1^)	0.07	0.12	354	0.068	36

S-YE was the sulfured organic fraction, such as cystine and methionine, present in 1 g L^−1^ of yeast extract. S-thiosulfate was the sulfur from thiosulfate present at 0.06 mol L^−1^ (2 mol of S per mole of thiosulfate). Cells represented the maximum concentrations obtained in the two growth conditions (data coming from Table 3). S-cells corresponded to the cellular sulfur. S-cells/S-(YE + thio) was the molar ratio of the cellular sulfur on the total of the yeast–sulfur and thiosulfate–sulfur

From these results, in the absence of thiosulfate, the cells incorporated 35% (Table 4) of the organic sulfur initially present in the yeast extract. Considering that organic sulfur is the growth-limiting element, it can be estimated that the remaining 65% of the S-YE are not accessible to the cells.

In the presence of thiosulfate, the calculations show that 36% of the initial sulfur (S-YE and S-thiosulfate) have been incorporated into the cellular material (Table 4). Two options concerning the origin of the S-cell can be considered. Either both the yeast extract and the thiosulfate contributed to the S-cell, or the thiosulfate was the only sulfur source. In the first case, if the assimilable S-YE was primarily incorporated into the biomass, the remaining S-cell would correspond to 36% of the initial S-thiosulfate. In the second case, 56% of the thiosulfate was incorporated into the cellular material. Therefore, in both cases, the fraction of the S-thiosulfate incorporated in the cellular material is lower than 56% of the initially present S-thiosulfate. Given the dissymmetry of the oxidation level of the two sulfurs in thiosulfate (S–SO_3_^−^), these observations would suggest that only one of the two sulfurs would actually be incorporated into the cellular material.

In Table 3, the analysis of carbon balances, obtained for various thiosulfate concentrations, showed that carbon recovery decreased drastically when thiosulfate concentrations limited *T. maritima* growth (e.g., inferior to 0.06 mmol L^−1^). In the absence of thiosulfate, the production of cells, lactate, acetate, and CO_2_ represented about only 54.9% of the carbon from the consumed glucose versus 78–87% for fermentation runs performed with more than 0.06 mmol L^−1^ of thiosulfate. The additional analyses of culture fermentation products, performed in the absence of thiosulfate and in the presence of 0.12 and 0.24 mmol L^−1^ of thiosulfate, revealed the presence of l-alanine (1.39, 3.8, and 3.8 mmol L^−1^, respectively) and extracellular polysaccharides (EPS) (4.7, 3.6, 2.9 mmol L^−1^ in equivalent glucose). Taking into account the carbon, represented by l-alanine and EPS, for these three culture conditions (0.0, 0.12, and 0.24 mmol L^−1^ of thiosulfate), the levels of C-recoveries (C^2^-recoveries) (87.2, 92.2, and 95.8%) concluded that almost all of the carbon from the fermented glucose was recovered. Under these three culture conditions, the production of l-alanine expressed as a percentage (c-alanine/fermented c-glucose) evolved almost constantly (between 4.2 and 4.6%). This result was entirely consistent with those published in the literature. Indeed, it should be noted that, in *T. maritima*, alanine production never represented more than 4–5% of the carbon from the consumed carbohydrate, whatever the evaluated growth conditions [43, 46, 49]. In contrast, EPS production, expressed as a percentage (C-EPS/C-glucose fermented), increased considerably in the absence of thiosulfate (28.6, 8.5, 7.0% versus 0.0, 0.12, and 0.24 mmol L^−1^ of thiosulfate, respectively). It should be noted that EPS are probably underestimated. Indeed, when preparing the samples for the EPS assay, the cultures were centrifuged to remove the cells which also eliminated the fraction of the EPS that is trapped with the cells. This EPS overproduction, revealed in the absence of thiosulfate, is not surprising since EPS could account for up to more than 20% of the carbon from the consumed carbohydrate depending on culture conditions [43, 46].

It must be emphasized that, in *T. maritima,* EPS production was associated with stress conditions such as oxidative stress or deficiency in ammonium [43, 45, 46]. Indeed, they should play a significant role in the defense strategy employed by this species and other anaerobes to cope with unfavorable environmental constraints [45, 46, 66, 67]. From this perspective, our results demonstrated, for the first time, that, in *T. maritima,* the deficiency of sulfur nutriment should be also considered as a stress condition marked by a stimulation of EPS production as an end-product of glucose fermentation.

In *T. maritima* cultures, performed under oxidative stress by oxygen [43] or under nutritional stress by sulfur nutriment deficiency (here the thiosulfate), both acetate and lactate yields were found to decline concomitantly in favor of the EPS yields. Interestingly, the major difference between patterns for the end-products of glucose fermentation in these two under-stress cultures was marked by the change in proportions between lactate and acetate molar yields. Indeed, *T. maritima* grown under oxidative stress by oxygen [43] showed an additional shift of glucose catabolism towards lactate, where 0.8 mol of lactate was produced per mol of acetate [43]. Although the limitation of *T. maritima* growth by thiosulfate was found to decrease drastically both acetate and lactate yields down to a low limit (0.7 and 0.3 mol mol^−1^, respectively in absence of thiosulfate), the proportion between these two molar yields remained however constant for all evaluated conditions (about 1 mol of lactate produced per 2 mol of acetate produced) (Table 3).

**Fig. 4:**
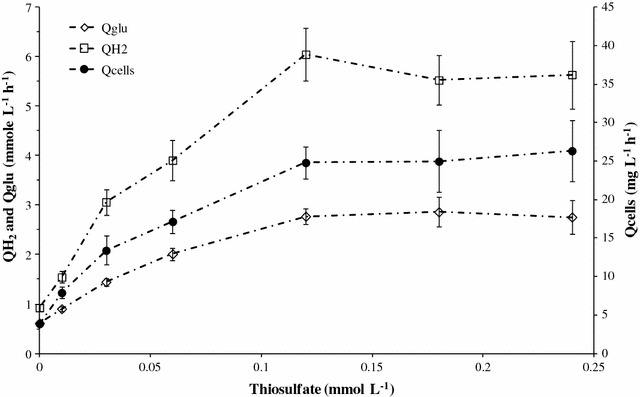
Glucose consumption and hydrogen and cells productivities versus thiosulfate concentrations. Hydrogen productivities (QH_2_) and glucose consumptions (Qglu) were expressed in mmol per liter of medium and per hour. *T. maritima* cells productivities (Qcells) were expressed in mg per liter of medium and per hour. These fermentation parameters were calculated during the growth phase from fermentation runs performed in bioreactor (in triplicate). For all fermentations, culture medium contained glucose (60 mmol L^−1^) and yeast extract (1 g L^−1^)

The studies addressing *T. maritima* metabolism have showed that this species harvested energy by glycolysis via the Embden–Meyerhof pathway (EMP) as the main route [39, 41]. For the ultimate steps of pyruvate reductions, acetate, H_2_, and CO_2_ (1:2:1 as molar proportions) represented the major end-products of glucose fermentation, whereas lactate, alanine, and EPS were minor. In agreement with this classical fermentation model, the H_2_ yield is only optimized when all glucose is converted to acetate. The highest H_2_ molar yield, that can be therefore achieved by this fermentation model, was 4 mol of H_2_ per mole of glucose (or other hexose) referred to as the Thauer limit [38, 39, 68, 69]. Under our experimental conditions, the results showed that all molar yields, including H_2_/glu, acetate/glu, and lactate/glu, declined sharply in thiosulfate-limited *T. maritima* growth (range 0–0.06 mmol L^−1^ of thiosulfate) (Table 3). In contrast, when thiosulfate was in excess (concentrations higher than 0.12 mmol L^−1^), the molar yields on glucose for acetate, lactate, and H_2_ reached 1–1.1, 0.4–0.5, and 2–2.2 mol mol^−1^ as upper limit, respectively (Table 3). Similarly, the kinetic patterns for the hydrogen specific productivity (q H_2_) and glucose specific consumption (q glucose) increased when the initial thiosulfate concentration increased (Table 3). The changes in the values of these two parameters indicated that the presence of thiosulfate accelerated the glucose consumption and, consequently, the hydrogen production to 18.8 and 40.4 mmol g^−1^ h^−1^ as upper limits, respectively (Table 3). In addition, consequently to the increases, all together of cellular concentration, cellular yield (cells/glu), and specific rates (q H_2_ and q glucose) (Table 3), the effect of thiosulfate was even greater on the volumetric hydrogen and cellular production, and volumetric glucose consumption (Qcells, QH_2_, and Qglu, respectively) (Fig. 4). As shown in the Fig. 4, Qcells and QH_2_ were increased sixfold and Qglu fourfold when thiosulfate concentrations were not limiting *T. maritima* growth (higher than 0.12 mmol L^−1^) in comparison to culture performed in absence of thiosulfate (Fig. 4). The upper limits for Qcells, QH_2_, and Qglu reached, in these cases, 25 mg L^−1^ h^−1^, 5.6, and 2.8 mmol L^−1^ h^−1^, respectively (Fig. 4).

Compared to the theoretical Thauer limit (4 mol mol^−1^), the relative weakness of the best H_2_ molar yields (H_2_/glu) (2–2.2 mol mol^−1^) (Table 3) obtained in this work is naturally explained by the nutrient constraints (excess of glucose with regard to the yeast extract), which had to be imposed in order to achieve the objectives of our study (effects of the concentration of thiosulfate on *T. maritima* growth). Nevertheless, these yields (2–2.2 mol mol^−1^) were coherent with the range of H_2_ molar yields (1.7–4.0 mol mol^−1^) reported in literature for *T. maritima* and *T. neapolitana* [16, 29, 39, 44, 53, 55, 68, 70–72]. It is the same for the highest QH_2_ (5.6 mmol L^−1^ h^−1^) (Fig. 3) which was also ranked in the middle of the range of QH_2_ values (1 to 14 mmol L^−1^ h^−1^) reported in literature [16, 29, 68, 70–72]. Thus, the comparison between the data in this study, for H_2_/glu, QH_2_, as well as cellular yields on glucose (cells/glu), with those found in the literature (8.5–9.6 g mol^−1^ of glucose, as in Table 3, versus 45 g mol^−1^ as reported by Schroder et al. [39]) suggested that, except for thiosulfate and glucose, the other compounds of our culture medium have to be optimized to reach the theoretical Thauer limit (4 mol of H_2_ per mole of glucose) and the maximum H_2_ productivity.

## Conclusions

This study has highlighted the necessary requirement, in a culture medium, of sulfur sources, including sulfide, cysteine, or thiosulfate, to grow *T. maritima*. The focus on thiosulfate, used as model, demonstrated that, in extremely controlled experimental conditions and with glucose as energy source, *T. maritima* growth was drastically limited by thiosulfate in the range of 0–0.06 mmol L^−1^. Under such experimental conditions, solely limited by thiosulfate, the cellular yield (*Y X/Thio*) was accurately determined (3617 ± 176 mg of cells per mmol of thiosulfate consumed). This evaluation was necessary to build a mathematical Monod-based model using glucose, yeast extract, and thiosulfate concentrations and the partial pressure of hydrogen as variables. This model, which simulates *T. maritima* growth and hydrogen production from glucose fermentation, will be published in the Part II of this publication [62].

Moreover, the results of this study showed that, under yeast extract-limited culture conditions, among all the nutrients present in the yeast extract, sulfur compounds, including both cystine (cysteine dimer) and methionine, were the ones limiting *T. maritima* growth.

So far, thiosulfate, as well as sulfur, was considered only as a detoxifying agent preventing the accumulation of H_2_ by oxidation into sulfide. This oxidation therefore relieves the inhibition by H_2_ of *T. maritima* and most *Thermotoga* species growth. This study underlined the increase of *T. maritima* growth with very low thiosulfate concentrations, for which the detoxifying effect is negligible. Instead, when its concentration was low, thiosulfate was found to be a sulfured nutriment required for the growth, forcing to reconsider its role in this species and most probably also in all thiosulfato-reducer hyperthermophiles. From now on, thiosulfate should be considered in *T. maritima* (1) as a sulfur source used for the synthesis of cellular materials (anabolism including proteins and Fe–S clusters dedicated to hydrogenase and ferredoxins, for instance) when thiosulfate is present at low concentrations (about 0.06 mmol L^−1^ under our experimental conditions), and (2) as both sulfur source and detoxifying agent at higher concentrations. Concerning this latter case, the intensity of the hydrogen detoxification function will depend on the thiosulfate availability and the level of the hydrogen partial pressure within culture medium as discussed in the part II of this manuscript at the end of “Results and discussion” section [62].

Otherwise, based on the comparison of the patterns for the end-products of glucose fermentation, obtained from *T. maritima* grown under different stress conditions such as nutritional deficiencies by nitrogen or thiosulfate, or oxidative stress by the presence of oxygen, amazing analogies were highlighted and discussed.

Finally, as demonstrated in this study, the thiosulfate addition, in the culture medium formulation, in a range of 0.12–0.24 mmol L^−1^ was able to increase significantly the cellular and hydrogen productivities in *T. maritima* by a factor of 6 compared to a thiosulfate-free medium. Based on these results, it can be recommended, for all works dealing with the optimization of hydrogen production from hexoses by dark fermentation using *T. maritima* or *T. neapolitana,* to add thiosulfate in the medium in the proportion of about 0.3 mmol of thiosulfate per g of cells (calculated from *Y X/thio* = 3617 mg of cells (cell dry weight) per mmol of thiosulfate). Thus, it will stimulate the cellular growth and hydrogen production while limiting the sulfide production within biogas, which is a damaging product for biotechnological applications in the energy field.

## Abbreviations

EPS: extracellular polysaccharides
EMP: Embden–Meyerhof pathway
ED: Entner–Doudoroff pathway
pH2: partial pressure of dihydrogen expressed in millibars
Eh: measurement, expressed in millivolts, of the reduction potential relative to a standard hydrogen electrode
DSMZ: Deutsche Sammlung von Mikroorganismen und Zellkulturen
DMSO: dimethyl sulfoxide
Na_2_S: sodium sulfide
micro-GC: micro gas chromatograph
GC-FPD: gas chromatograph flame photometric detector
OD: optical density
CDW: cell dry weight
HPLC: high Pressure liquid chromatograph
Qcells: cellular production rate [mg (cdw) L^−1^ h^−1^)]
Qglu: glucose consumption rate (mmol L^−1^ h^−1^)
QH_2_: hydrogen production rate (mmol L^−1^ h^−1^)
